# MicroRNA-449a delays lung cancer development through inhibiting KDM3A/HIF-1α axis

**DOI:** 10.1186/s12967-021-02881-8

**Published:** 2021-05-27

**Authors:** Shan Hu, Peng Cao, Kangle Kong, Peng Han, Yu Deng, Fan Li, Bo Zhao

**Affiliations:** Department of Thoracic Surgery, Tongji Hospital, Tongji Medical College, Huazhong University of Science and Technology, Qiaokou District, No. 1095 Jiefang Avenue, Qiaokou District, Wuhan, 430030 Hubei China

**Keywords:** Lung cancer, MicroRNA-449a, Lysine demethylase 3A, Hypoxia-induced factor-1α

## Abstract

**Background:**

It has been established that microRNA (miR)-449a is anti-tumorigenic in cancers, including lung cancer. Therefore, this study further explored miR-449a-mediated mechanism in lung cancer, mainly focusing on lysine demethylase 3A/hypoxia-induced factor-1α (KDM3A/HIF-1α) axis.

**Methods:**

miR-449a, KDM3A and HIF-1α levels in lung cancer tissues and cell lines (A549, H1299 and H460) were measured. Loss- and gain-of-function assays were performed and then cell proliferation, cell cycle, apoptosis, invasion and migration were traced. The relationship between KDM3A, miR-449a and HIF-1α was verified. Tumor growth in vivo was also monitored.

**Results:**

Both lung cancer tissues and cells exhibited reduced miR-449a and raised KDM3A and HIF-1α levels. miR-449a interacted with KDM3A; HIF-1α could bind with KDM3A. Up-regulating miR-449a hindered while suppressing miR-449a induced lung cancer development via mediating HIF-1α. Elevating KDM3A promoted cellular aggression while down-regulating KDM3A had the opposite effects. Up-regulating KDM3A or HIF-1α negated up-regulated miR-449a-induced effects on cellular growth in lung cancer. Restoring miR-449a impaired tumorigenesis in vivo in lung cancer.

**Conclusion:**

It is eventually concluded that miR-449a delays lung cancer development through suppressing KDM3A/HIF-1α axis.

**Supplementary Information:**

The online version contains supplementary material available at 10.1186/s12967-021-02881-8.

## Background

Lung cancer is the major human malignancy, mainly composed of small cell lung cancer and non-small cell lung cancer (NSCLC) [1]. It is usually diagnosed as advanced in which lung cancer cells quickly pass through the lymph or blood and spread to other organs, causing a high mortality globally [2]. Statistically, more than twice as many men as women have been diagnosed with lung cancer worldwide, which is mainly due to the risk factor, smoking [3]. Most lung cancer patients, especially those in an advanced stage, have poor prognosis due to delayed diagnosis and poor response to conventional treatments [4]. Though received treatments, up to 15% of lung cancer patients develop second primary lung cancer [5]. At present, the pathogenesis of lung cancer is not completely comprehended that asks for in-depth explorations.

Dysregulated non-coding RNAs, including microRNAs (miRNAs) can affect the occurrence and metastasis of lung cancer through transcriptional, post transcriptional and epigenetic activities [6]. miR-449a is a part in regulating lung cancer proliferation, migration, invasion and epithelial to mesenchymal transition [7]. Clinically analyzed, low expression of miR-449a is closely connected with differentiation, clinical staging and lymph node metastasis of lung cancer patients [8]. In treating lung cancer, miR-449a has the potential to overcome the resistance of epidermal growth factor receptor tyrosine kinase inhibitors [9]. Also, targeting miR-449a could sensitize non-small sell lung cancer cells to ionizing radiation, thereby suppressing tumor growth [10]. Lysine demethylase 3A (KDM3A), also known as JMJD1A, is the overexpressed gene in lung cancer [11]. KDM3A is the vital modulator for biological functions of NSCLC cancer cells [12]. Mechanistically, KDM3A could induce the production of inhibitory cytokines in lung adenocarcinoma cells, thereby promoting immune escape of cancer cells [13]. Serving as a hypoxic response gene, KDM3A could be mediated by hypoxia-inducible factor-1α (HIF-1α), the key gene in lung cancer under a hypoxic microenvironment [14]. The stability of HIF-1α has been recognized to modulate the stem-like characteristics of lung cancer cells [15], as well as drug resistance and tumor progression in lung cancer [16]. Regarding to the functional roles of miR-449a, KDM3A and HIF-1α in lung cancer, this research was initiated to decode whether their integrity involved in lung cancer and targeting miR-449a/KDM3A/HIF-1α axis regulated cancer progression.

## Methods and materials

### Ethics statement

Approved by the ethics committee of Tongji Hospital, Tongji Medical College, Huazhong University of Science and Technology, this study was conducted following *Declaration of Helsinki*. An signed consent was provided by each participant. Animal treatment was humanely performed.

### Sample collection

Lung cancer tissues and normal tissues were harvested from 80 patients (not received radiotherapy or chemotherapy) in Tongji Hospital, Tongji Medical College, Huazhong University of Science and Technology from April 2018 to March 2019. All tissues were frozen in liquid nitrogen. The diagnosis was conducted by the pathologists in Tongji Hospital, Tongji Medical College, Huazhong University of Science and Technology in compliance with the principles of the latest classification regulations of the World Health Organization [17].

### Cell culture

Human lung cancer cell lines (A549, H1299 and H460) and normal human lung epithelial cell line (BEAS-2B) were supplied by the Cell Bank of Chinese Academy of Sciences (Shanghai, China) and maintained in 10% fetal bovine serum (FBS)-Dulbecco's modified Eagle medium (DMEM) (both from HyClone, Los Angeles, USA) [18].

### Cell transfection

Based on the known miR-449a and KDM3A sequences in NCBI, plasmids were constructed by Sangon Biotech (Shanghai, China). Cells of passage 3 were trypsinized and cultured into a monolayer in a 24-well plate at 1 × 10^3^ cells/well. Cells at 75% confluence were transfected with miR-449a-mimic/inhibitor, sh-KDM3A, overexpression (oe)-KDM3A, miR-449a-mimic + oe-HIF-1α, miR-449a-mimic + oe-KDM3A, or their corresponding negative control (NC) through Lipofectamine 2000 (Thermo Fisher Scientific, Waltham, USA) [19].

### Cell counting kit (CCK)-8 assay

Cells in a 96-well plate (1 × 10^3^ cells/well) were cultured for 0, 24, 48, and 72 h, respectively. At each time point, each well was supplemented with 10 μL CCK-8 reagent (Dojindo, Kumamoto, Japan). Cells that were continuously cultured for further 3 h were detected by a microplate reader (Infinite M200 PRO, TECAN, Switzerland) to measure the optical density (OD_450 nm_) [18].

### Transwell assay

Cells (6 × 10^4^ cells) were resuspended in serum-free DMEM (200 μL), and added in the chamber. An attractant was set with 10% FBS-DMEM (600 μL) in the lower chamber. Cells were incubated for 24 h in migration assay and for 48 h in invasion assay. Cells that migrated or invaded were fixed with 4% paraformaldehyde, followed by staining with 0.1% crystal violet and photography by a microscope (TE2000; Nikon, Tokyo, Japan) in at least five random fields of view [20].

### Flow cytometry

A549 cells transfected for 48 h were pre-cooled with 70% frozen ethanol and treated with propidium iodide (PI) and RNase (BD Company, New Jersey, USA). Cell cycle distribution was analyzed with a flow cytometer (BD Biosciences, New Jersey, USA) [21]. For cell apoptosis, cells were detached with 0.25% trypsin, added with Roswell Park Memorial Institute-1640 medium containing 10% FBS, centrifuged at 1000 r/min, fixed with 70% ethanol and adjusted to 1 × 10^6^ cells/mL. The cell suspension was added with 10 mL Annexin V-fluorescein isothiocyanate (FITC)/PI (556,547, Shanghai Shuojia Biotechnology Co., Ltd., China) and cell apoptosis was detected in a flow cytometer (XL type, Conlter, USA). Excitation wavelength was 480 nm, FITC was detected at 530 nm and PI was detected at greater than 575 nm. The apoptotic rate was percentage of apoptotic cells in total cells [22].

### Reverse transcription quantitative polymerase chain reaction (RT-qPCR)

RNA was extracted from tissues and cells by Trizol (Life Technologies, MD, USA). cDNA was generated by a reverse transcription kit (Thermo, Massachusetts, USA). miR-449a, KDM3A, and HIF-1α levels were measured by SYBR Green method on the ABI7500 fluorescent quantitative PCR instrument, and normalized to U6 and β-actin. Data analysis was conferred to 2^−ΔΔCt^ method. Primers were found in Table 1 [23].

**Table 1:** Primer sequences

Primer sequences	Forward (5’ → 3’)	Reverse (5’ → 3’)
miR-449a	CTCGCTGGCAGTGTATTGTTAG	TATCGTTGTACTCCAGACCAAGAC
KDM3A	AACTATTGAGCCACACAGACAGG	ACACATACTCCAAACCCACACC
HIF-1α	GGTTCCAGCAGACCCAGTTA	AGGCTCCTTGGATGAGCTTT
U6	CTCGCTTCGGCAGCACA	AACGCTTCACGAATTTGCGT
β-actin	CATCACCATCTTCCAGGAGCG	TGACCTTGCCCACAGCCTTG

*miR-449a* microRNA-449a, * KDM3A* Lysine demethylase 3A, * HIF-1α* Hypoxia-induced factor-1α

### Western blot assay

The extracted total protein in cells and tissues by radio-immunoprecipitation assay (RIPA) buffer was quantified by bicinchoninic acid protein assay kit (Thermo Fisher Scientific). Subsequently, total protein was separated with 10% sodium dodecyl sulfate–polyacrylamide gel electrophoresis and transferred onto a 0.45-µM polyvinylidene fluoride membrane (MilliPore, MA, USA). The protein membrane incubated with the specific antibodies were developed by enhanced chemiluminescence and exposed by Image Quant LAS 4000C (GE Company, USA). Primary antibodies included KDM3A (1:200, 12,835–1-AP, Proteintech), HIF-1α (1:1000, 610,958, BD Biosciences), Cleaved-PARP (ab32064, Abcam, MA, USA), Cleaved-CASP3 (9661, Cell Signaling Technology) and β-actin (1:1000, sc-47778, Santa Cruz Biotechnology) while secondary antibody included anti-rabbit immunoglobulin G (IgG; 7074, 1:2000, Cell Signaling Technology). β-actin was the internal control [24].

### Dual luciferase reporter gene assay

The binding sites of miR-449a and KDM3 were firstly analyzed by RNA22 and their targeting relation was validated by dual luciferase reporter gene experiment. KDM3A mRNA 3'untranslated region (3'UTR) and the mutant sequence were cloned into the pGL3 dual luciferase reporter gene vector (Promega, WI, USA) to construct wild-type and mutant KDM3A 3'UTR vectors. The constructed vectors with miR-449a mimic/NC-mimic were co-transfected into A549 cells through Lipofectamine 2000 (Invitrogen). The luciferase activity was measured by the dual luciferase reporter system (Glomax20/20, ATCC, Manassas, VA, USA) [25].

### Co-immunoprecipitation (Co-IP) assay

Cells were lysed with IP lysis buffer (500 μL) on ice and centrifuged at 13,500 r/min. The supernatant was reacted with the antibody overnight, added with protein A + G agarose (P2055, Beyotime, China) and centrifuged at 13,500 r/min. After washing 3 times with cold lysis buffer, the immunoprecipitate was combined with sodium dodecyl sulfate, heated to 98 °C and treated with sodium dodecyl sulphate polyacrylamide gel electrophoresis. After that, the protein was transferred to a 0.22-μm polyvinylidene fluoride membrane, immersed in 10% skimmed milk and combined with the primary antibodies KDM3A (1:200, 12,835–1-AP, Proteintech) and HIF-1α (1:1000, 610,958, BD Biosciences). Then, the membrane was washed 3 times with Tris-buffered saline with Tween-20 and cultured with IgG antibody (7074, 1:2000, Cell Signaling Technology). The pellet was rinsed with RIPA buffer and then subjected to Western blot analysis. Normal IgG was used as a NC [26].

### Tumor xenografts in nude mice

Twenty-five BALA/C nude mice (4–6 weeks old, 18–20 g) from Hunan SJA laboratory Animal Co., Ltd. (Changsha, China) were subcutaneously injected with A549 cells (1 × 10^3^ cells) into the right armpit. Phosphate-buffered saline (PBS)-treated A549 cells were used as NC. The average diameter of tumors was measured weekly. After 8 w, all mice were euthanized and tumor weight was measured [27].

### Statistical analysis

The independent t-test was applied to compare data between two groups. All data were analyzed by statistical software SPSS 17.0 (SPSS, Chicago, USA) and expressed as mean ± standard deviation. *P* < 0.05 was considered a significant difference.

## Results

### Down-regulated miR-449a is manifested in lung cancer tissues and cells

miR-449a expression has been measured to reduce in primary lung cancer [28]. Similar to that we applied RT-qPCR to detect miR-449a level in lung cancer and ultimately discovered that miR-449a was lowly expressed in lung cancer tissues and cells (Additional file 1: Fig. S1a, b). Among lung cancer cell lines, lowest miR-449a level was detected in A549 and H1299 cells, thus A549 and H1299 cells were used miR-449a overexpression experiments.

### Over-expressing miR-449a delays lung cancer development while down-regulating miR-449a has the opposite effects on lung cancer

**Fig. 1:**
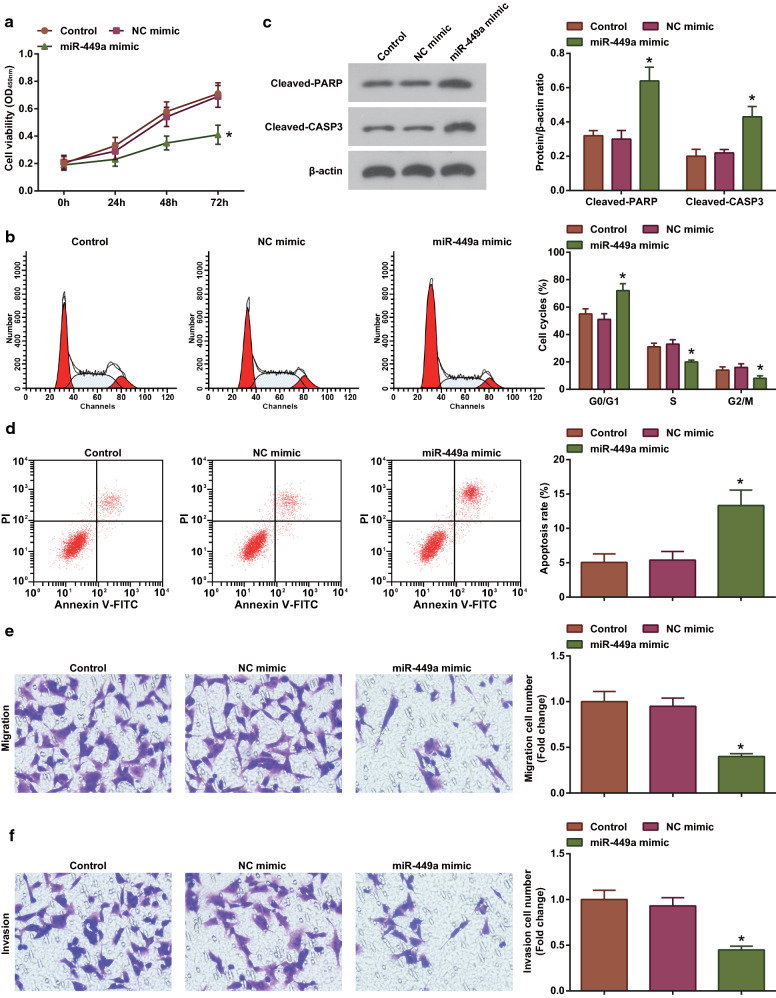
Over-expressing miR-449a delays A549 cell growth. **a** CCK-8 assay analyzed A549 cell proliferation after overexpression of miR-449a; **b** Flow cytometry analyzed A549 cell cycle after overexpression of miR-449a; **c** Western blot analyzed the expression of apoptosis-related proteins in A549 cells after overexpression of miR-449a; **d** Flow cytometry analyzed A549 cell apoptosis after overexpression of miR-449a; **e** Transwell assay analyzed A549 cell migration after overexpression of miR-449a; **f** Transwell assay analyzed A549 cell invasion after overexpression of miR-449a; The data from three independent experiments were expressed as mean ± standard deviation. *P < 0.05 compared with the NC-mimic group

To further clarify the effect of miR-449a on lung cancer, cell proliferation, apoptosis/cell cycle distribution, migration and invasion, as well as apoptosis-related proteins (Cleaved-PARP and Cleaved CASP3) were examined by CCK-8, flow cytometry, Transwell assay and western blot, respectively. The experimental results demonstrated that in response to up-regulation of miR-449a in A549 (Additional file 1: Fig. S1c), proliferation, migration and invasion capacities were impaired, apoptosis was enhanced, increased cells arrested in G0/G1 phase, and Cleaved-PARP and Cleaved CASP3 protein expression were elevated (Fig. 1a–f).

**Fig. 2:**
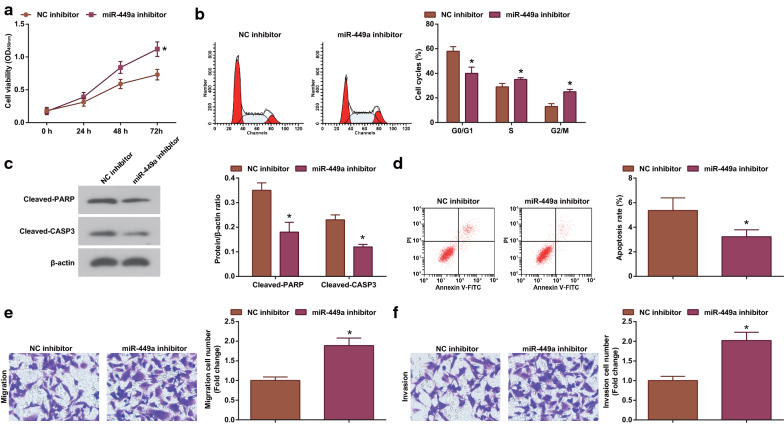
Suppressing miR-449a accelerates lung cancer development. **a** CCK-8 assay analyzed A549 cell proliferation after inhibiting miR-449a; **b** Flow cytometry analyzed A549 cell cycle after inhibiting miR-449a; **c** Western blot analyzed the expression of apoptosis-related proteins in A549 cells after inhibiting miR-449a; **d** Flow cytometry analyzed A549 cell apoptosis after inhibiting miR-449a; **e** Transwell assay analyzed A549 cell migration after inhibiting miR-449a; **f** Transwell assay analyzed A549 cell invasion after inhibiting miR-449a; The data from three independent experiments were expressed as mean ± standard deviation. * *P* < 0.05 compared with the NC-inhibitor group

To make the research results more rigorous, we repeated the above experiments in another lung cancer cell line H1299, and the results were similar to those in A549 cells (Additional file 3: Fig. S3). Moreover, we performed miR-449a down-regulation assay A549 cells (Additional file 1: Fig. S1d), and found that down-regulating miR-449a enhanced the growth of A549 cells (Fig. 2a–f).

HIF-1α, generally highly expressed in lung cancer cells, is considered as the main mediator of lung cancer cells under hypoxia [29, 30]. We determined HIF-1α level in lung cancer tissues and normal tissues and tested a higher level of HIF-1α in lung cancer tissues (Additional file 2: Fig. S2a). HIF-1α level presented the same trend in lung cancer cells in vitro (Additional file 2: Fig. S2b). Subsequently, the effect of miR-449a on HIF-1α was explored, and the result indicated that overexpressing miR-449a reduced HIF-1α level (Additional file 2: Fig. S2c) while silencing miR-449a raised HIF-1α expression in cells (Additional file 2: Fig. S2d).

### Enhanced KDM3A level in lung cancer cells promotes cellular aggression

**Fig. 3:**
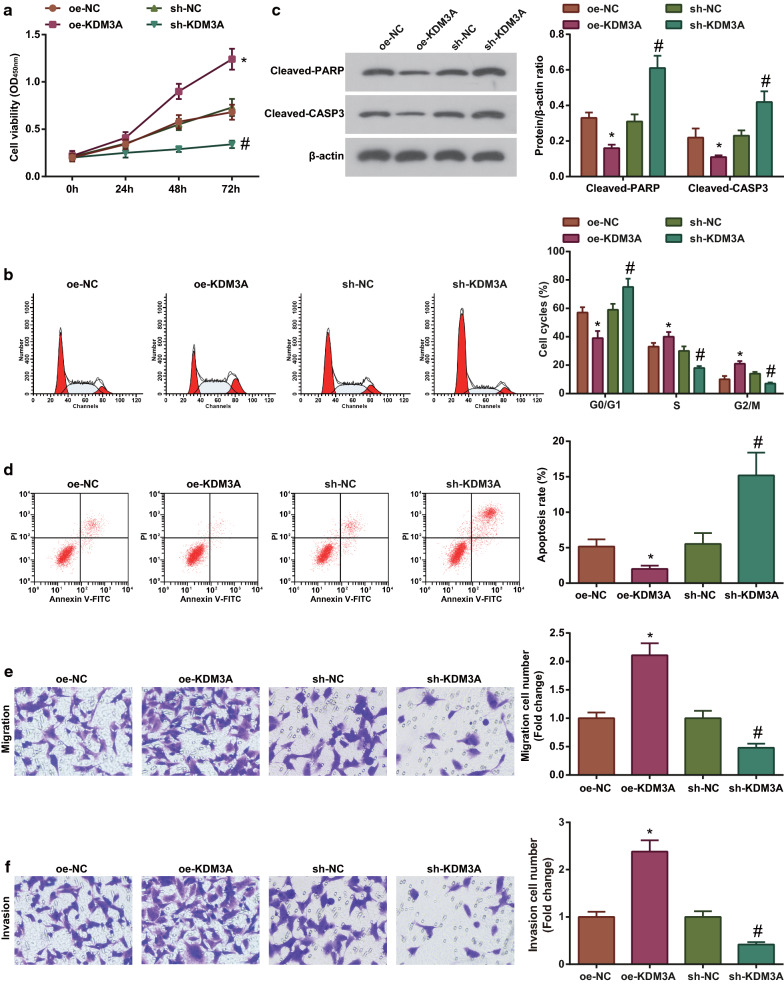
Elevating KDM3A promotes cellular aggression in lung cancer while down-regulating KDM3A has the opposite effects. **a** CCK-8 assay analyzed A549 cell proliferation after interference with KDM3A; **b** Flow cytometry analyzed A549 cell cycle after interference with KDM3A; **c** Western blot analyzed the expression of apoptosis-related proteins in A549 cells after interference with KDM3A; **d** Flow cytometry analyzed A549 cell apoptosis after interference with KDM3A; **e** Transwell assay analyzed A549 cell migration after interference with KDM3A; **f** Transwell assay analyzed A549 cell invasion after interference with KDM3A; The data from three independent experiments were expressed as mean ± standard deviation. *P < 0.05 compared with the oe-NC group. #*P* < 0.05 compared with the sh-NC group

KDM3A is the mediator of biological progress in cancers and is up-regulated in lung cancer [13, 31]. We also tested KDM3A expression in lung cancer tissues and cells, and found that KDM3A showed an increase in both lung cancer tissues and cells (Additional file 1: Fig. S1e, f). To study the effects of KDM3A in lung cancer, we transfected oe-KDM3A and sh-KDM3A plasmids into A549 cells to up-regulate or down-regulate KDM3A expression (Additional file 1: Fig. S1g). Then, we observed cellular growth and tested that cell growth was promoted after oe-KDM3A treatment while suppressed after sh-KDM3A treatment (Fig. 3a–f). Shortly, up-regulating KDM3A promoted while down-regulating KDM3A suppressed the development of lung cancer.

### miR-449a interacts with KDM3A; HIF-1α could bind to KDM3A

**Fig. 4:**
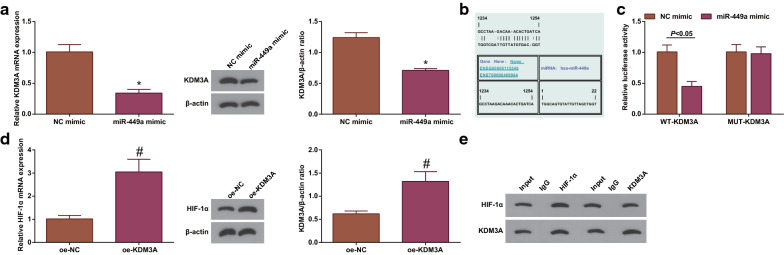
miR-449a interacts with KDM3A; HIF-1α could bind with KDM3A. **a** RT-qPCR and Western blot analyzed KDM3A expression in cells after overexpression of miR-449a; **b** Bioinformatics website predicted the binding sites of miR-449a and KDM3A; **c** Luciferase reporter gene experiment verified the relation between miR-449a and KDM3A; **d** RT-qPCR and Western blot analyzed HIF-1α expression in cells after overexpression of KDM3A; **e**. CO-IP assay analyzed the interaction between KDM3A and HIF-1α; The data from three independent experiments were expressed as mean ± standard deviation. *P < 0.05 compared with the the NC-mimic group; #*P* < 0.05 compared with the the oe-NC group

miR-449a and KDM3A have been recorded to regulate lung cancer, respectively [17, 29]. Focused on the issue that whether KDM3A was involved in the process of miR-449a regulating lung cancer, we firstly determined the effect of miR-449a on KDM3A expression and found that overexpressing miR-449a decreased KDM3A level in cells (Fig. 4a). After that, the targeting relation was analyzed between miR-449a and KDM3A on Jefferson website (Fig. 4b) and validated by dual luciferase reporter experiment. It turned out that miR-449a-mimic weakened luciferase activity of WT-KDM3A (Fig. 4c).

HIF1α and KDM3A are positively correlated [32, 33]. However, how KDM3A regulates the expression of HIF1α is still unclear. Thus, we conducted assays to explore the interaction between the two. First, HIF-1α expression was measured after up-regulating KDM3A and it was found that HIF-1α level was indeed enhanced by KDM3A overexpression (Fig. 4d). Furthermore, a two-way CO-IP test was performed and the result reflected that KDM3A and HIF-1α were co-precipitated in A549 cells by anti-KDM3A antibody (Fig. 4e). It was suggested that HIF-1α could bind to KDM3A.

### Up-regulating KDM3A or HIF-1α negates the overexpressed miR-449a-induced suppression on cellular growth in lung cancer

**Fig. 5:**
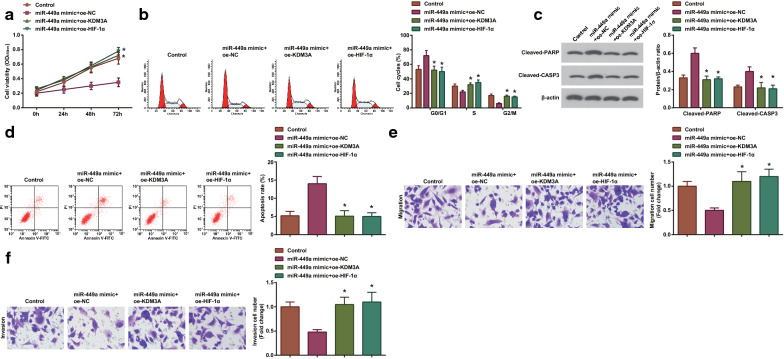
Up-regulating KDM3A or HIF-1α negates up-regulated miR-449a-induced suppression on cellular growth in lung cancer. **a** CCK-8 analyzed A549 cell proliferation in the miR-449a-mimic + oe-NC group, miR-449a-mimic + oe-KDM3A group, and miR-449a-mimic + oe-HIF-1α group; **b** Flow cytometry analyzed A549 cell cycle in these three groups; **c** Western blot analyzed the expression of apoptosis-related proteins in A549 cells in these three groups; **d** Flow cytometry analyzed A549 cell apoptosis in these three groups; **e** Transwell assay analyzed A549 cell migration in these three groups; **f** Transwell assay analyzed A549 cell invasion in these three groups; The data from three independent experiments were expressed as mean ± standard deviation. *P < 0.05 compared with the the miR-449a-mimic + oe-NC group

Though we have confirmed the effects of miR-449a and KDM3A on lung cancer, and identified the targeting relation between miR-449a and KDM3A, as well as between KDM3A and HIF-1α, the internal mechanism of miR-449a/KDM3A/HIF-1α axis is not yet clear. Therefore, a co-transfection system was conducted: up-regulation of KDM3A or HIF-1α was performed simultaneously with overexpression of miR-449a. Then, it was revealed that restoring KDM3A or HIF-1α negated overexpressed miR-449a-induced effects on cellular growth in lung cancer (Fig. 5a–f). Moreover, miR-449a-mediated suppression on HIF-1α expression was impaired by up-regulation of KDM3A (Additional file 2: Fig. S2e). Thus, it could be concluded that elevating KDM3A or HIF-1α negated up-regulated miR-449a-induced suppression on cellular growth in lung cancer.

### Restoring miR-449a impairs tumorigenesis in vivo in lung cancer

**Fig. 6:**
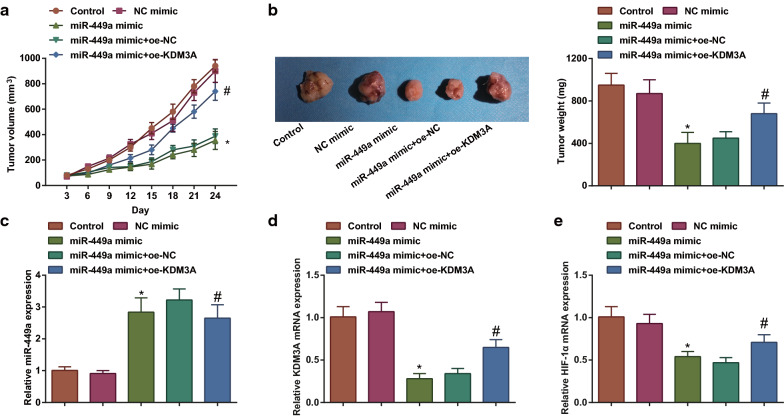
Restoring miR-449a impairs tumorigenesis in vivo in lung cancer. **a** Tumor volume in mice after tumor xenografts; **b** Tumor weight in mice after tumor xenografts; **c** RT-qPCR analyzed miR-449a expression in xenografted tumors; **d** RT-qPCR analyzed KDM3A expression in xenografted tumors; E. RT-qPCR analyzed HIF-1α expression in xenografted tumors; n = 5. The data were expressed as mean ± standard deviation. *P < 0.05 compared with the NC-mimic group; #*P* < 0.05 compared with the miR-449a-mimic + oe-NC group

A549 cells that had been transfected with the plasmids were injected into mice to study whether miR-449a/KDM3A/HIF-1α axis regulated lung cancer progression in vivo. It was measured that up-regulating miR-449a decreased tumor volume and weight. However, KDM3A overexpression impaired such anti-tumor effects of miR-449a on mice with lung cancer (Fig. 6a, b). In addition, miR-449a expression was increased whereas KDM3A and HIF-1α levels were decreased in xenografted tumors treated with up-regulated miR-449a, which were reversed by enhanced expression of KDM3A (Fig. 6c–e). Shortly, miR-449a/KDM3A/HIF-1α axis regulated lung cancer tumor growth in vivo.

## Discussion

Lung cancer remains the major cancer type in male and female with enigmatic pathogenesis [34]. In the present paper, a part of the underlying mechanism in lung cancer was explained from the axis of miR-449a/KDM3A/HIF-1α. We determined a low expression of miR-449a in lung cancer and further figured out that over-expressing miR-449a delayed while suppressing miR-449a accelerated lung cancer development in vitro and in vivo, which was related to its negative regulation on HIF-1α. Besides, KDM3A, validated as the target of miR-449a, was overexpressed in lung cancer and promoted cellular aggression. Collectively, miR-449a depressed KDM3A to incur tumor activities in lung cancer through down-regulating HIF-1α.

miR-449a has been regarded as a tumor suppressor in cancers, whose functional overexpression could hamper cancer cell growth. The repressed level of miR-449a is also detected in NSCLC, and up-regulated miR-449a can damage proliferation, migration and invasion of cancer cells whereas down-regulated miR-449a functions in an opposite way [7]. As suggested in a late research, the proliferative and anti-apoptotic behaviors of lung cancer cells are limited by enhanced miR-449a, and greatly impaired by ultrasound-microbubble-mediated miR-449a [8]. The overexpressed miR-449a has been witnessed to negatively mediate A aisintegrin and metalloproteinases 10, thereby weakening the migratory and invasive capacities of NSCLC cells [35]. Through targeting high mobility group box 1, miR-449a sets the obstacle for NSCLC cells to proliferate, migrate and invade in vitro and to form tumors in vivo [18]. Further proved by a published report, lung cancer cells containing up-regulated miR-449a behave less aggressively and mainly arrest in G1/G0 phase [36]. Transient up-regulation of miR-449a in lung cancer cells functions to promote cell senescence and that in xenograted tumors depresses tumor growth, which is related the regulatory mechanism with E2F transcription factor 3 [17]. From those previous reports, it was confirmed that miR-449a was repressive for lung cancer development.

Though KDM3A was studied as a target of miR-449a in the present study, their targeting relation has not been widely validated in other researches, which needs further verification in proceeding explorations. Increased level of KDM3A has been shown in lung cancer and artificially silencing KDM3A obtains the ability to damage cell proliferation and migration [29]. In a former research focusing on NSCLC, the aberrantly elevated KDM3A expression manifests in cancer tissues and knocking down KDM3A attributes to the suppression on cell biological functions [12]. Not limited to lung cancer, the contributory effects of KDM3A overexpression are detected in other cancers. Exampled by a recent report, the invasive ability of breast cancer cells manifesting inhibited KDM3A is impaired in hypoxia [37]. KDM3A level can be elevated by hypoxia in pancreatic cancer and the incremental expression of KDM3A could stimulate cancer development whereas its suppression had the opposite effects [38]. Of interest, KDM3A restoration is the base to acquire an anti-apoptotic phenotype of myeloma cell apoptosis in chronic hypoxia through accumulating HIF-1α [39].

Widely investigated, the up-regulated HIF-1α exhibits in lung cancer [40, 41]. The down-regulated HIF-1α in NSCLC cells is a part contributing to the repressive effects of methanol-ethyl acetate partitioned fraction from Magnolia grandiflora on tumor cell invasion and migration [42]. HIF-1α down-regulation is capable of preventing cellular growth and stimulating cell apoptosis in NSCLC [43]. HIF-1α level can be suppressed by up-regulating miR-199a, thereafter devoting to NSCLC cell proliferation suppression [44]. The depletion of long non-coding RNA cancer susceptibility 9 suppressively modifies the proliferation, invasion and migration of lung cancer, which is connected with its positive feedback loop with HIF-1α [45].

### Conclusion

On the whole, it is summarized that miR-449a interacts with KDM3A/HIF-1α axis, and performs anti-tumor effects on lung cancer. From the axis of miR-449a/KDM3A/HIF-1α, the molecular mechanism of lung cancer has been further comprehended, and this axis may alight the potentials to treat lung cancer. Limitations to relative small experimental scale ask for further confirmation of the results in a larger study size.

## Supplementary Information

**Additional file 1: Figure S1** RT-qPCR and Western blot analysis of miR-449a and KDM3A expression. **a** RT-qPCR analyzed miR-449a expression in lung cancer tissues and normal tissues, n = 80; **b** RT-qPCR analyzed miR-449a expression in normal human lung epithelial cell lines BEAS-2B and lung cancer cell lines (A549, H1299 and H460); **c** RT-qPCR analyzed miR-449a expression in A549 cells after overexpression of miR-449a; **d** RT-qPCR analyzed miR-449a expression in A549 cells after inhibiting miR-449a; **e** RT-qPCR and Western blot analyzed KDM3A expression in lung cancer tissues and normal tissues, n = 80; **f**. RT-qPCR and Western blot analyzed KDM3A expression in normal human lung epithelal cell line BEAS-2B and lung cancer cell lines (A549, H1299 and H460); **g** RT-qPCR and Western blot analyzed KDM3A expression in A549 cells after interference with KDM3A; The data from three independent experiments were expressed as mean ± standard deviation.* *P* < 0.05 compared with the oe-NC group. # *P* < 0.05 compared with the sh-NC group.

**Additional file 2: Figure S2** RT-qPCR and Western blot analysis of HIF-1α expression. **a** RT-qPCR analyzed HIF-1α expression in lung cancer tissues and normal tissues, n = 80; **b** RT-qPCR analyzed HIF-1α expression in normal human lung epithelial cell line BEAS-2B and lung cancer cell lines (A549, H1299 and H460); **c** RT-qPCR and Western blot analyzed HIF-1α expression in A549 cells after overexpression of miR-449a; **d** RT-qPCR and Western blot analyzed HIF-1α expression in cells after inhibiting miR-449a; **e** RT-qPCR and Western blot analyzed HIF-1α expression in these three groups; The data from three independent experiments were expressed as mean ± standard deviation. *P < 0.05 compared with the NC-mimic group. # *P* < 0.05 compared with the NC-inhibitor group. & *P* < 0.05 compared with the the miR-449a-mimic + oe-NC group.

**Additional file 3: Figure S3** Overexpression of miR-449a suppresses the growht of H1299 cells. **a** RT-qPCR analyzed miR-449a expression in H1299 cells after overexpression of miR-449a; **b** CCK-8 assay analyzed H1299 cell proliferation after overexpression of miR-449a; **c** Flow cytometry analyzed H1299 cell cycle after overexpression of miR-449a; **d** Western blot analyzed the expression of apoptosis-related proteins in H1299 cells after overexpression of miR-449a; **e** Flow cytometry analyzed H1299 cell apoptosis after overexpression of miR-449a; **f** Transwell assay analyzed H1299 cell migration after overexpression of miR-449a; **g** Transwell assay analyzed H1299 cell invasion after overexpression of miR-449a; The data from three independent experiments were expressed as mean ± standard deviation. *indicates *P* < 0.05 compared with the NC-mimic group.

## Data Availability

Not applicable.

## Abbreviations

miR: MicroRNA
KDM3A/HIF-1α: Lysine demethylase 3A/hypoxia-induced factor-1α
NSCLC: Non-small cell lung cancer
miRNAs: MicroRNAs
FBS: Fetal bovine serum
CCK: Cell counting kit
RT-qPCR: Reverse transcription quantitative polymerase chain reaction
GAPDH: Glyceraldehyde-3-phosphate dehydrogenase
3'UTR: 3'Untranslated region
Co-IP: Co-immunoprecipitation
